# Electronic, Thermal and Mechanical Properties of Carbon and Boron Nitride Holey Graphyne Monolayers

**DOI:** 10.3390/ma16206642

**Published:** 2023-10-11

**Authors:** Bohayra Mortazavi

**Affiliations:** Department of Mathematics and Physics, Leibniz Universität Hannover, Appelstraße 11, 30167 Hannover, Germany; bohayra.mortazavi@gmail.com

**Keywords:** holey graphyne, boron nitride, machine learning, first-principles, monolayers

## Abstract

In a recent experimental accomplishment, a two-dimensional holey graphyne semiconducting nanosheet with unusual annulative π-extension has been fabricated. Motivated by the aforementioned advance, herein we theoretically explore the electronic, dynamical stability, thermal and mechanical properties of carbon (C) and boron nitride (BN) holey graphyne (HGY) monolayers. Density functional theory (DFT) results reveal that while the C-HGY monolayer shows an appealing direct gap of 1.00 (0.50) eV according to the HSE06(PBE) functional, the BNHGY monolayer is an indirect insulator with large band gaps of 5.58 (4.20) eV. Furthermore, the elastic modulus (ultimate tensile strength) values of the single-layer C- and BN-HGY are predicted to be 127(41) and 105(29) GPa, respectively. The phononic and thermal properties are further investigated using machine learning interatomic potentials (MLIPs). The predicted phonon spectra confirm the dynamical stability of these novel nanoporous lattices. The room temperature lattice thermal conductivity of the considered monolayers is estimated to be very close, around 14.0 ± 1.5 W/mK. At room temperature, the C-HGY and BN-HGY monolayers are predicted to yield an ultrahigh negative thermal expansion coefficient, by more than one order of magnitude larger than that of the graphene. The presented results reveal decent stability, anomalously low elastic modulus to tensile strength ratio, ultrahigh negative thermal expansion coefficients and moderate lattice thermal conductivity of the semiconducting C-HGY and insulating BN-HGY monolayers.

## 1. Introduction

Graphene [1,2,3], the most stable two-dimensional (2D) form of carbon atoms is a highly attractive nanomaterial because of exhibiting exceptional mechanical strength [4], phononic and carrier mobilities [5,6,7] and interesting electro-optical characteristics [8,9,10,11]. Nonetheless, this wonderful nanosheet is not a semiconductor with an appropriate band gap, and therefore, its application in nanotransistors and nanosensors becomes limited. A promising strategy for tailoring graphene’s electronic bandgap is to manipulate its atomic structure by introducing tetragonal, pentagonal, heptagonal or octagonal rings [12,13,14,15,16]. Amidst diverse 2D full-carbon nanomaterials, the sp and sp^2^ hybridized graphyne and graphdiyne nanoporous lattices [17] show outstanding physical properties, such as finite-gap semiconducting electronic characters [18], critically needed for proficient applications in opto-electronics, nanosensors and catalytic nanodevices. In contrast with graphene, which can be straightforwardly derived from the mechanical exfoliation of bulk graphite or chemical vapor deposition over various metallic substrates, the synthesis of graphyne and graphdiyne lattices is conspicuously more complicated and challenging. In 2010, Li and coworkers [19] pioneered the fabrication of graphdiyne through a cross-coupling reaction. Subsequently, Matsuoka et al. [20] achieved a significant breakthrough in 2017 by employing an innovative chemical reaction method for graphdiyne synthesis. These pivotal advancements paved the way for further progress in this realm, leading Kan et al. [21], Wang et al. [22] and Matsuoka et al. [23] to successfully fabricate nitrogen-, boron- and triphenylene-based-graphdiyne nanomembranes, respectively. 

In the last decade, significant strides have been made in creating diverse graphdiyne nanosheets, but the production of graphyne structures has presented more formidable hurdles. In a noteworthy development in the realm of all-carbon 2D structures, in 2022, Hu et al. [24] achieved a breakthrough by successfully crafting layered materials of γ-graphyne through a reversible dynamic alkyne metathesis of alkynyl-substituted benzene building blocks. In another exciting advance in the field of carbon 2D nanosheets, Liu and colleagues [25], with the aid of an interfacial Castro–Stephens-type coupling reaction using 1,3,5-tribromo-2,4,6-triethynylbenzene, could for the first time produce the full carbon holey graphyne (C-HGY). From the practical point of view, it is very insightful to evaluate the stability and various physical properties of the C-HGY nanosheets. Another interesting aspect is whether the boron nitride holey graphyne (BN-HGY) counterpart could be stable and if yes, what are the resulting physical properties. In this work, a combination of first-principles density functional theory (DFT) calculations and machine-learning-based classical modeling were conducted to evaluate the dynamical stability, the electronic, mechanical, thermal expansion and lattice thermal conduction properties of the suspended and defect-free C- and BN-HGY monolayers. 

## 2. Computational Methods

The Vienna ab initio simulation package (VASP) [26,27] was employed for DFT calculations with Perdew–Burke–Ernzerhof (PBE) within the generalized gradient approximation (GGA) and a kinetic energy cutoff of 500 eV. Because of the nanoporous structures, Grimme’s DFT-D3 [28] van der Waals (vdW) dispersion correction was adopted to better account for the interactions of non-bonded atoms. Optimization of both lattice parameters and atomic positions was conducted using the conjugate gradient method, adopting a 3 × 3 × 1 Monkhorst-Pack [29] k-point grid and energy and force convergence criteria of 10^−5^ eV and 0.002 eV/Å, respectively. To prevent vdW interactions along the thickness, a 15 Å vacuum spacing was employed. Moment tensor potentials (MTPs) [30] were fitted to investigate the phononic and thermal properties with the aid of the MLIP package [31]. Ab initio molecular dynamics (AIMD) simulations were carried out in order to acquire the training dataset with a time step of 1 fs, DFT-D3 and 2 × 2 × 1 grid over stress-free and uniaxially unit cells under varying temperatures, similar to our recent works [32,33]. MTPs with a cutoff distance of 3.5 Å were trained using the two-step passive training approach used in our recent studies [32,33,34]. The phonon dispersion relation was obtained using the developed MTPs, over 5 × 5 × 1 supercells, and by adopting the small displacement method of the PHONOPY package [35], as explained in our earlier investigation [36]. We utilized the LAMMPS package [37] to examine the thermal properties based on the developed MTPs. Non-equilibrium molecular dynamics (NEMD) simulations were carried out to evaluate the lattice thermal conductivity of the C- and BN-HGY monolayers, using the same approach as that adopted in our previous work [34].

## 3. Results and Discussions

**Figure 1:**
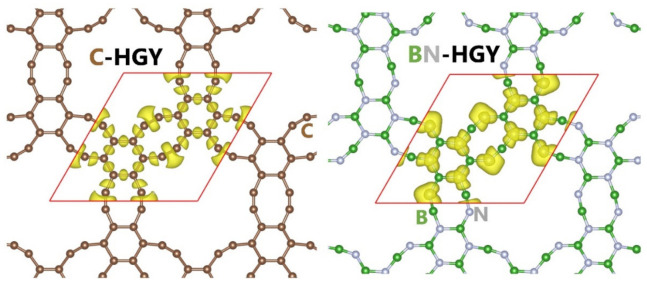
Top view of the C- and BN-HGY monolayers’ fully planar crystal structures along with the ELF isosurface of 0.75 plotted within the unit cell area using the VESTA [40] software. Here, the horizontal and vertical directions represent X and Y directions, respectively.

We first investigated the structural features of the experimentally realized C-HGY [25] and herein theoretically predicted BN-HGY monolayers, which are illustrated in Figure 1. The C- and BN-HGY are topographically completely flat structures, which perfect hexagonal lattices and optimized lattice constants of 10.845 and 10.968 7 Å, respectively. These novel lattices are made of periodically benzene rings linked by triple C≡C bonds, exhibiting patterns of six-vertex and elongated eight-vertex rings. The predicted lattice constant of the C-HGY matches excellently with recent reports [25,38]. To examine the nature of the interactions in C- and BN-HGY lattices, electron localization function (ELF) [39] with an isosurface value of 0.75 is also shown in Figure 1. The appearance of large electron localization over 0.75 around the middle of bonds indicates that the C-C and B-N bonds in this system are covalent. It is also evident that because of the higher electronegativity of the nitrogen compared with boron atoms, the ELF is pronounced over the former atom in the BN-HGY lattice. The atomic structure of the energy minimized lattices are included in the Supporting Information Document.

**Figure 2:**
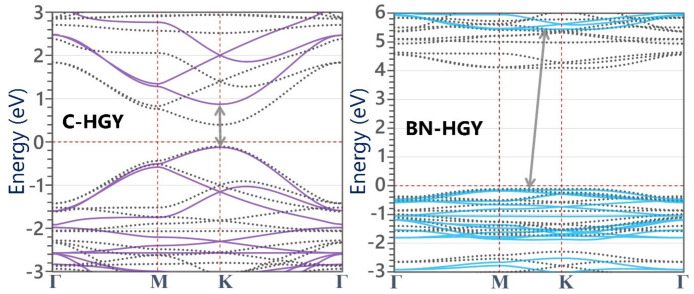
HSE06- (continuous lines) and PBE/GGA- (dotted lines) based electronic band structures of (**left**) C- and (**right**) BN-HGY monolayers. The Fermi level is set to 0 eV. The arrows show the position of band gap within the HSE06 method.

In order to investigate the electronic properties of the C- and BN-HGY monolayers, we carried out the electronic band structure calculations using PBE/GGA and HSE06 functionals, which are illustrated in Figure 2. The C-HGY monolayer is found to be a direct gap semiconductor with band gaps of 1.00 (0.50) eV, according to the HSE06(PBE) functional occurring at point K. The predicted electronic band gaps of the C-HGY monolayer match excellently with a recent theoretical report [38]. In contrast with the native C-HGY lattice, the BN-HGY monolayer is found to be an indirect gap insulator with a large band gaps of 5.58 (4.20) eV, according to the HSE06(PBE) methods. These findings are consistent with the semimetallic and insulating electronic nature of pristine and suspended graphene and h-BN native lattices. In the BN-HGY monolayer, the valance band maximum (VBM) and conduction band minimum (CBM) appear with almost flat dispersions, which suggest low carrier mobilities and high insulating features of this system. In the original C-HGY lattice, nonetheless, the VBM and CBM appear with rather sharp dispersions, which have been proven to result in outstanding carrier mobilities [25,38]. 

**Figure 3:**
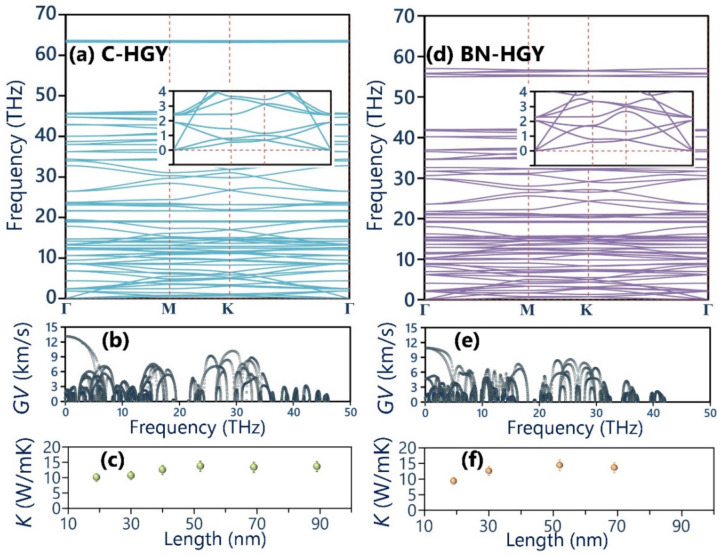
(**a**) Phonon dispersion relations of the (**a**) C- and (**d**) BN-HGY monolayers; here, insets show the zoomed view of low-frequency phonon modes. (Predicted phonons’ group velocity (*GV*) of the single-layer (**b**) C- and (**e**) BN-HGY. (**c**,**f**) Length-dependent phononic thermal conductivity (*κ*) the room temperature.

Having analyzed the structural and electronic characteristics, we now shift our consideration to the analysis of the thermal properties of the C- and BN-HGY monolayers. In Figure 3, the predicted phonon dispersion curves of these novel lattices with the aid of the fitted MLIPs are presented, which reveals that none of the three acoustic phonon and entire optical branches exhibit imaginary frequencies, indicating the desired dynamical stability of the suspended monolayers. As shown in Figure 3, insets of phonon band structures, from the point where the three acoustic phonon modes originate, in which the out-of-plane (ZA) shows quadratic relations, and the other two in-plane counterparts appear with nearly linear dispersions, in analogy with those in the single-layer graphene and most of 2D materials [36,41]. The in-plane longitudinal acoustic phonon modes show the sharpest dispersions, which reveal their largest phonon group velocities of 13.1 and 10.8 km/s, along the C- and BN-HGY monolayers, respectively, as shown in Figure 3. The transverse acoustic phonon modes show considerably lower maximum group velocities of around 2.7 and 5.2 km/s for the C- and BN-HGY monolayers, respectively. It is noticeable that the ZA acoustic modes exhibit narrow dispersions, which reveal their remarkably suppressed phonon group velocities, consistent with the results shown in Figure 3. In addition, except the ZA mode, significant crossing is exposed for the all phonon bands throughout the whole frequency range, revealing remarkable scattering rates and short lifetimes for the majority of phononic energy carriers in the C- and BN-HGY nanosheets. From the presented phonon dispersion relation, short lifetimes can be concluded for the phonons in these nanomembranes, suggesting rather low lattice thermal conductivity, despite the existence of strong covalent interactions. In Figure 3, the MLIP-based NEMD predictions for the length effects on the room temperature phononic thermal conductivity of the C- and BN-HGY monolayers are plotted, assuming a fixed thickness of the 3.6 Å for both lattices, according to the experimental report [25]. Consistent with our preliminary findings, the room temperature lattice thermal conductivity of the considered monolayers both converged to a rather low value of around 14.0 ± 1.5 W/mK. It appears that the higher group velocity of the transverse acoustic mode in the BN-HGY system compensates for the lower group velocity of the longitudinal counterpart, as compared with the original C-HGY lattice. It is worth noting that using a combination of the Boltzmann transport equation and DFT method, Sajjad et al. [38] predicted a thermal conductivity of 29 W/mK at 300 K for the C-HGY monolayer, which is almost twice as high as our own estimation. In their work, the authors accounted for no more than three-phonon scattering, which might significantly overstate the thermal conductivity. One advantage of the NEMD method is that it inherently considers multi-phonon scatterings, and can thus provide more realistic estimations. The trained MTPs and atomic structures in the LAMMPS input formats are given in the Supporting Information Document.

**Figure 4:**
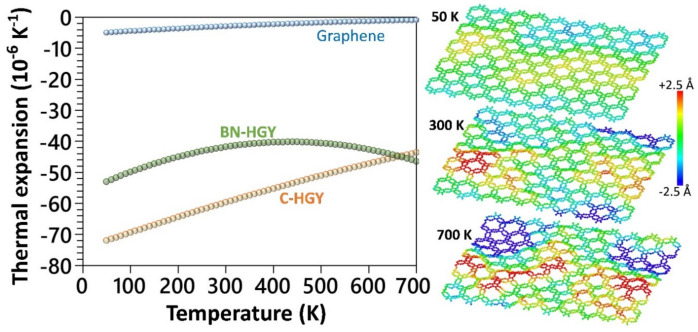
MLIP-based molecular dynamics results for the thermal expansion coefficients of the suspended C-HGY, BN-HGY and graphene [42] monolayers as a function of temperature. The atomic structures show the side views of the C-HGY monolayer equilibrated at 50, 300 and 700 K, with color coding representing the out-of-plane displacements of atoms.

We now examine the thermal expansion behavior using the developed MLIP-based classical models. The temperature dependency of the thermal expansion coefficients was evaluated based on the $\frac{1}{A}\frac{dA}{dT}$ relation, where *A* denotes the projected area at a given temperature of *T* [42,43]. To obtain the aforementioned relation, six independent calculations with uncorrelated initial velocities were carried out over systems with 2400 atoms, and subsequently for each structure the projected areas at every temperature were averaged and a polynomial curve was fitted to report the thermal expansion coefficients [42]. The details of the MTP-based molecular dynamics calculations are fully given in our earlier study [42]. In Figure 4, the thermal expansion coefficients of the single-layer C- and BN-HGY are compared with those of the graphene [42]. The thermal expansion coefficients of the suspended C-HGY, BN-HGY and graphene monolayers at room temperature were predicted to be −59.7, −41.8 and −2.9 × 10^−6^ K^−1^ [42], respectively, which shows one order of magnitude larger negative thermal expansion of these novel 2D lattices. As illustrated in Figure 4, for the atomic structures of the C-HGY lattice at 50, 300 and 500 K, it is noticeable that at low temperatures, this nanosheet presents a fairly flat configuration; however, at higher temperatures, due to presence of nanoporous lattice and low bending rigidity, it undergoes substantial out-of-plane wrinkles. The substantial formation of wrinkles at higher temperatures results in the shrinkage of the projected area of the sheet and subsequently, appears with ultrahigh negative thermal expansion coefficients [42]. 

**Figure 5:**
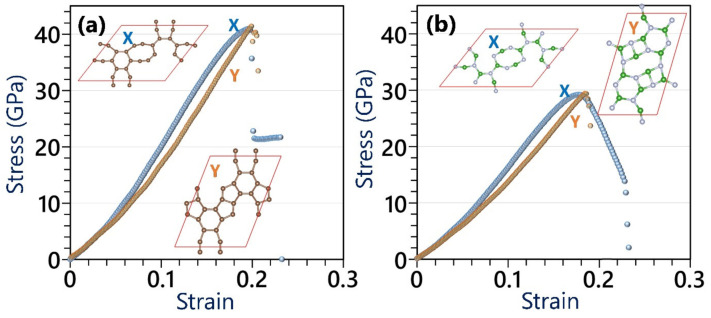
Comparison of the uniaxial stress–strain curves of the (**a**) C- and (**b**) BN-HGY monolayers by the ground state DFT under loading along X and Y directions. Insets show the atomic configurations after passing the ultimate tensile strength point.

We finally explore the mechanical properties of the C- and BN-HGY monolayers using the DFT method, assuming a constant thickness of 3.6 Å during the entire mechanical loading, to report the stress values in the GPa unit. In Figure 5, the uniaxial stress–strain curves of the considered lattices are plotted. The elastic modulus was acquired by fitting linear curves to stress–strain values under 0.01 stress. According to the presented DFT results, although marginal mismatches are observable in the stress–strain curves along the X and Y directions, both systems exhibit convincingly isotropic elasticity and ultimate tensile strength. The elastic modulus (ultimate tensile strength) values of the single-layer C- and BN-HGY are predicted to be 127(41) and 105(29) GPa, respectively. It is worth mentioning that according to the Griffth theory [44], the elastic modulus to ultimate tensile strength ratio should be around 10, which for the pristine graphene, MoS_2_ and silicene monolayers was reported to be 8 [4], ~11 [45], and ~9 [46], respectively. As an interesting behavior, the aforementioned ratio for the C- and BN-HGY monolayers is around 3. In Figure 5 insets, we also compared the failure mechanism, which revealed that along the X direction, the first bond breakages occur within hexagonal rings’ bonds, oriented exactly along the loading on the boundary of nanopore, suddenly suppressing the load bearing ability of the systems and resulting in conspicuous sharp stress drops. On the other side, for both considered lattices under uniaxial loading along the Y direction, as it can be seen from Figure 5 insets, the structural transformations occur after passing the tensile strength point (strain levels around 0.2), resulting in structural instabilities and lack of clear bond breakages. 

## 4. Concluding Remarks

Inspired by the recent experimental synthesis of carbon holey graphyne (C-HGY) [25], herein we explored the electronic, dynamical stability, thermal and mechanical properties of C- and BN-HGY monolayers using the state-of-the-art theoretical calculations. The main findings can be summarized as follows:

(a) Predicted phonon dispersion curves confirm the desired dynamical stability of the suspended C- and BN-HGY monolayers.
(b) The C-HGY monolayer is a direct gap semiconductor with 1.00 (0.50) eV gaps, according to the HSE06(PBE) functional.
(c) The BN-HGY monolayer is predicted to be an insulator with low carrier mobilities due to almost flat dispersions of VBM and CBM.
(d) The room temperature lattice thermal conductivity of the suspended C- and BN-HGY nanosheets is estimated to be very close, around 14.0 ± 1.5 W/mK.
(e) The thermal expansion coefficient of the C- and BN-HGY nanomembranes at room temperature are predicted to be by more than one order of magnitude larger than that of the graphene.
(f) The elastic modulus (ultimate tensile strength) values of the single-layer C- and BN-HGY are predicted to be 127(41) and 105(29) GPa, respectively.
(g) The elastic modulus to ultimate tensile strength ratio of these nanoporous lattices at the ground state is predicted to be around 3, which is almost one third of that of other prominent 2D materials.

## Data Availability

Atomic structure of the energy-minimized lattices in the VASP and LAMMPS input formats, and the fitted MLIPs are included in the Supporting Information Document. Additional data presented in this study are also available on request from the corresponding author.

## Supplementary Materials

The following supporting information can be downloaded at: https://www.mdpi.com/article/10.3390/ma16206642/s1.
